# Relationships between anopheline mosquitoes and topography in West Timor and Java, Indonesia

**DOI:** 10.1186/1475-2875-9-242

**Published:** 2010-08-26

**Authors:** Ermi Ndoen, Clyde Wild, Pat Dale, Neil Sipe, Mike Dale

**Affiliations:** 1 Griffith School of Environment, Griffith University, Queensland, Australia; 2 Environmental Futures Centre, Griffith University, Queensland, Australia; 3 Urban Research Program, Griffith University, Queensland, Australia

## Abstract

**Background:**

Malaria is a serious health issue in Indonesia. Mosquito control is one aspect of an integrated malaria management programme. To focus resources on priority areas, information is needed about the vectors and their habitats. This research aimed to identify the relationship between anopheline mosquitoes and topography in West Timor and Java.

**Methods:**

Study areas were selected in three topographic types in West Timor and Java. These were: coastal plain, hilly (rice field) and highland. Adult mosquitoes were captured landing on humans identified to species level and counted.

**Results:**

Eleven species were recorded, four of which were significant for malaria transmission: *Anopheles aconitus, Anopheles barbirostris, Anopheles subpictus *and *Anopheles sundaicus*. Each species occupied different topographies, but only five were significantly associated: *Anopheles annularis, Anopheles vagus *and *Anopheles subpictus *(Java only) with hilly rice fields; *Anopheles barbirostris, Anopheles maculatus *and *Anopheles subpictus *(West Timor only) with coastal areas.

**Conclusion:**

Information on significant malaria vectors associated with specific topography is useful for planning the mosquito control aspect of malaria management.

## Background

Malaria is a life-threatening disease in Indonesia with an estimated 15 million cases and 42,000 deaths annually [1-3]. Malaria is transmitted by *Anopheles *mosquitoes. Of the 24 *Anopheles *species recorded in Indonesia, ten are major malaria vectors [4-6]. These include: *Anopheles aconitus*, *Anopheles balabacensis*, *Anopheles barbirostris*, *Anopheles farauti*, *Anopheles koliensis*, *Anopheles letifer*, *Anopheles maculatus*, *Anopheles punctulatus*, *Anopheles subpictus *and *Anopheles sundaicus *[5]. The most extensively occurring malaria vectors in Indonesia are *An. sundaicus*, *An. subpictus*, *An. barbirostris*, *An. maculatus*, *An. aconitus*, and *An. balabacensis *[5,7]. *Anopheles sundaicus *is associated with coastal, brackish water and is widely distributed from Sumatra through Java to Bali [8]. On Sumatra, however, it is regularly associated with fresh water [9]. Species recorded as important in Java include: *An. subpictus *[10,11]; *An. aconitus*, associated with rice-paddies, [8,10,12] and, in Central Java, according to Barcus *et al *[13], *An. sundaicus *is the dominant vector in coastal areas, and *An. aconitus *and *An. barbirostris *are often found in the coastal plains area. In hilly areas, *An. maculatus *and *An. balabacensis *are dominant. In Kalimantan, *An. balabacensis *is associated with forest [14] and is also recorded in western Sumbawa Island [15]. *Anopheles punctulatus, An. farauti and An. koliensis *are the main vectors of malaria in Papua [16,17].

Habitat preferences of the immature stages are indicative of the areas in which adult mosquitoes will be active. The larval wetland habitat may be temporary or permanent, and natural or man-made, and each species is associated with specific habitats [18].

Many of the factors important to mosquito development and survival, such as meteorological conditions, vegetation, water body characteristics and land use may be related to topography (mainly landform and elevation) and Sandosham and Thomas [19] suggested that *Anopheles *habitats may be divided into three main topographic zones: brackish-water zone, coastal plain, and hills and mountains.

### Aim of the research

The aim was to characterize the relationships between anopheline species and topography in West Timor and Java, Indonesia.

## Methods

### Study areas

**Figure 1:**
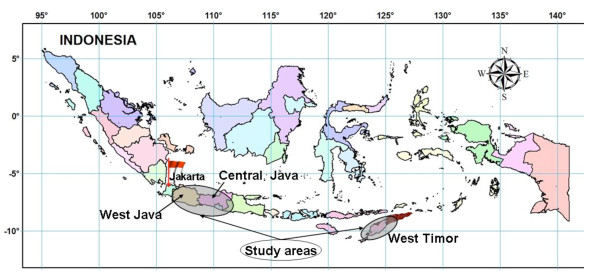
Location of study areas

Ten villages (five in West Timor and five in Java) were chosen to represent different types of topography adapted from the habitat information provided by Sandosham and Thomas [19]. These were coastal plain, hilly (rice fields), and highland areas. The villages were selected based also on their pattern of malaria, the availability of local assistants and access constraints. The areas were in West Timor, West Java and Central Java (together referred to as Java) as shown in Figure 1. The villages, selected by topographic types and land use, are shown in Table 1.

**Table 1:** Summary of adult mosquito surveys in West Timor and Java (West and Central Java)

Location (village)	Topographic type (and land use)	Altitude (m)	No of surveys		No of Collector-Nights	Dates of survey
			
			Nights	Collectors		
**WEST TIMOR**						

Noelbaki	Coastal - rice field	20	2	6	12	21-Jun, 5-Jul 2006

Tablolong	Coastal	10	2	6	12	20-Jul, 18-Aug 2006

Liufeto-Tuadale	Coastal, flat, rice field, lakes	15	3	6	18	2-Aug, 31-Aug, 28-Dec 2006

Sikumana	Hilly - rice field	198	6	6	36	29-Jun, 13-Jul, 27-Jul, 24-Aug, 12-Sep 06, 05-Jan 2007

Haumenbaki	Highland - rice field	739	12	4	48	25-Jun, 07-Jul, 21-Jul, 3-Aug, 25-Aug, 21-Sep, 19-Oct, 3-Nov, 17-Nov, 30-Nov, 7-Dec, 14-Dec 2006

**Total West Timor**			**25**	**28**	**126**	

**JAVA**						

**West Java - Sukabumi**						

Lodji	Coastal	35	4	6	24	30-Nov, 13-Dec, 28-Dec 2006, 12-Jan 2007
Kertajaya	Coastal	25	4	6	24	1-Dec, 12-Dec, 27-Dec 2006, 9-Jan 2007

Langkap Jaya, Lengkong	Highland	725	4	6	24	27-Nov, 11-Dec, 27-Dec 2006, 12-Jan 2007

	Total West Java		12	18	72	

**Central Java - Kebumen**						

Sadang Kulon	Hilly - rice field	144	6	4	24	18-Jan, 2-Feb, 17-Feb, 3-Mar, 17-Mar, 31-Mar 2007

Jojogan	Hilly - rice field	146	6	4	24	18-Jan, 02-Feb, 17-Feb, 3-Mar, 17-Mar, 31-Mar 2007

**Total Central Java**			12	8	48	

**Total Java**			**24**	**26**	**120**	

West Timor has one of the highest incidences of malaria in Indonesia [3]. In Java malaria is not considered to be endemic in Sukabumi District, although there was a malaria outbreak in 2003 [20,21]. In Kebumen, Central Java, there are several persistent pockets of malaria transmission [13,22,23], and malaria incidence in the Kebumen District is high relative to other locations in Central Java [24].

There is very little seasonal variation in climate of the study areas. However local meteorological conditions are important to mosquito activity. Maximum and minimum temperature, relative humidity and wind speed were checked during the study periods to ensure that conditions were suitable for mosquito activity.

### Mosquito surveys

A total of 49 mosquito surveys were conducted from June 2006 to March 2007; 25 in West Timor and 24 in Java (12 in West Java, Sukabumi and 12 in Central Java, Kebumen). Table 1 summarizes the surveys.

The adult captures were from landing rates. This was considered preferable to light trapping for two reasons. First, it would capture host-seeking mosquitoes (important to malaria transmission) and second, it did not require special equipment. Adult mosquito landing collections were conducted indoors and outdoors in the villages in each topographic type. The surveys were conducted fortnightly, based on the method recommended by WHO [25,26], and described below.

Adult mosquitoes were caught when landing on human volunteers' bare legs or hands during the night. For this research, ethical clearance was obtained from Indonesian Ministry of Health. Four to six people (depending on the location) were employed to catch these host-seeking mosquitoes. The collectors were divided into indoor and outdoor groups. Both indoor and outdoor catches were carried out generally from 6 pm to 6 am at one-hour intervals. The landing collection was conducted for forty minutes, followed by ten minutes collecting mosquitoes resting on the wall in the house, and outdoor-resting mosquitoes in animal enclosures. The last ten minutes were used for collectors to rest and change positions. To avoid collector bias, each collector was rotated into different location on an hourly basis. The collectors were equipped with torches: aspirators and paper cups were used to catch and store the mosquitoes.

The collections were identified to species level based on the identification key to female Anophelines of Indonesia provided by the Indonesian Ministry of Health [4]. The data were recorded as frequencies for each species by location and topographic type.

### Analyses

Chi-squared analyses was used to test the relationship between mosquito numbers, location (Java or West Timor) and topographic type (coastal plain, hilly, highland).

## Results

Although highland areas were slightly cooler, the differences in meteorological conditions were not so great that they would inhibit mosquito development or flight. The mean minimum temperature was 21.8°C (SD 3.4), mean maximum was 26.3°C (SD 2.7); mean minimum relative humidity was 71% (SD 9) and the mean maximum was 87% (SD 5). Wind speed was less than 2 ms^-1^.

Table 2 summarizes the numbers of mosquitoes caught for the eleven species by location (West Timor or Java). Of these, four species are considered to be especially important malaria vectors [5]: *Anopheles aconitus*, *An. barbirostris*, *An. subpictus *and *An. sundaicus*. A total of 7,189 mosquito individuals were caught, of which 77% were in Java, with eleven species present. The remaining 23% were in West Timor with large numbers of *An. subpictus *(an important vector).

**Table 2:** Summary of the results of the adult mosquito collections (species considered important for malaria transmission are in bold)

Surveys and species	West Timor		Java		Grand total	
	**Total**	**%**^**1**^	**Total**	**%**^**1**^	**Mosquitoes**	**%**

Total number of surveys	25		24		50	

Total collector-nights	126		120		246	

Number of *Anopheles *mosquitoes by species						

***An. aconitus***	10	0.6	1100	19.8	1110	15.4

*An. annularis*	16	1.0	489	8.8	505	7.0

***An. barbirostris***	247	15.2	518	9.3	765	10.6

*An. flavirostris*	0	0.0	4	0.1	4	0.1

*An. indefinitus*	0	0.0	481	8.6	481	6.7

*An. kochi*	0	0.0	9	0.2	9	0.1

*An. maculatus*	6	0.4	88	1.6	94	1.3

***An. subpictus***	1065	65.5	415	7.5	1480	20.6

***An. sundaicus***	0	0.0	35	0.6	35	0.5

*An. tesselatus*	0	0.0	4	0.1	4	0.1

*An. vagus*	282	17.3	2420	43.5	2702	37.6

Total mosquitoes captured	1626	100	5563	100	7189	100

Average per night	65		231.8		143.8	

Average per night per collector	4		11.6		3.8	

^1 ^The percentage off all mosquitoes captured at this location belonging to each species

Table 3 shows the Chi-squared results. The null hypothesis is rejected in five cases and the research hypothesis that there is a significant relationship between a particular species and topographic type is accepted.

**Table 3:** Relationships between *Anopheles *species and topographic types in West Timor and Java

Species	Frequency of observed Mosquitoes				Independence Comparison			*Remark*
		
	Location	Coastal	Hilly-rice field	Highland	**χ**^**2**^	df	p	
*An. aconitus*	West Timor	0	10	0	2.64	2	0.267	Not significant
	Java	50	870	180				

***An. annularis***	**West Timor**	**5**	**11**	**0**	**154**	**2**	**<0.000**	**Significant**
	**Java**	**0**	**489**	**0**				

***An. barbirostris***	**West Timor**	**220**	**19**	**8**	**359**	**2**	**<0.000**	**Significant**
	**Java**	**128**	**0**	**390**				

*An. flavirostris*	West Timor	0	0	0	--	2	--	--
	Java	0	4	0				

*An. indefinitus*	West Timor	0	0	0	--	2	--	--
	Java	0	481	0				

*An. kochi*	West Timor	0	0	0	--	2	--	--
	Java	0	9	0				

***An. maculatus***	**West Timor**	**0**	**1**	**5**	**40**	**2**	**<0.000**	**Significant**
	**Java**	**81**	**1**	**6**				

***An. subpictus***	**West Timor**	**1,065**	**0**	**0**	**1,480**	**2**	**~0**	**Significant**
	**Java**	**0**	**415**	**0**				

*An. sundaicus*	West Timor	0	0	0	--	2	--	--
	Java	35	0	0				

*An. tesselatus*	West Timor	0	0	0	--	2	--	--
	Java	1	3	0				

***An. vagus***	**West Timor**	**66**	**197**	**19**	**316**	**2**	**<0.000**	**Significant**
	**Java**	**112**	**2,308**	**0**				

the use of a dash (--) indicates that the contingency table was too sparse to permit analysis.

### Species

*Anopheles annularis*: this species was numerous in Java but not in West Timor. In Java it was strongly associated with hilly rice fields.

*Anopheles barbirostris: *in Java this species was strongly associated with highland areas, whereas in West Timor it was associated with coastal areas.

*Anopheles maculatus*: this was found almost exclusively in coastal areas of West Timor.

*Anopheles subpictus*: this species was found in Java but only in hilly rice fields, while it was predominant in coastal areas of West Timor

*Anopheles vagus*: this species was predominantly found in hilly rice field areas of Java and it was also associated, with fewer numbers, with coastal areas. It was not common in West Timor though its distribution was widespread.

The other species showed no significant relationships as identified by the analyses. This was related to relatively small numbers (leading to low expected values) for *An. flavirostris*, *Anopheles indefinus*, *Anopheles kochi*, *An. sundaicus *and *Anopheles tesselatus*. *Anopheles aconitus*, while a significant malaria vector, showed no relationship because it appears to be widespread and indiscriminate in its habitats.

### Location

In West Timor, *An. subpictus *was the most abundant species (65.5%), and was found only in coastal areas (Table 2). Species with widespread distribution from coastal to highland areas in West Timor were *An. barbirostris *and *An. vagus *(Table 3). In Java the most abundant species were *An. vagus *and *An. aconitus *(Table 3). *Anopheles maculatus *and *An. sundaicus *only occurred in coastal areas, whereas *An. annularis*, *An. indefinitus*, *An. kochi *and *An. subpictus *were captured only in hilly rice field areas.

### Topographic type

**Figure 2:**
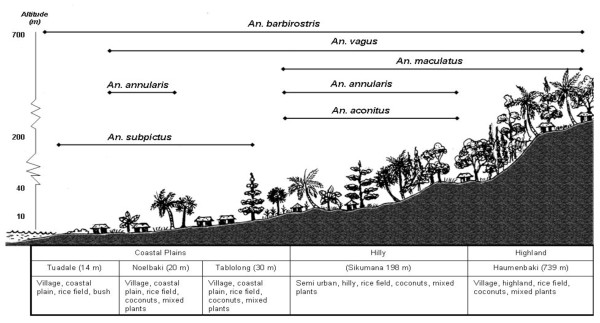
Schematic summary of distribution of anopheline mosquitoes relative to topographic and land use characteristics in West Timor

**Figure 3:**
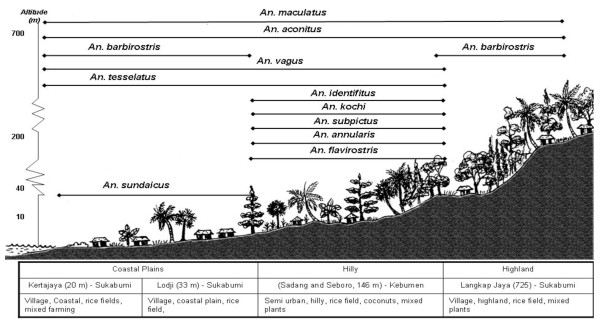
Schematic summary of distribution of anopheline mosquitoes relative to topographic and land use characteristics in Java

In terms of topographic type coastal areas were the preferred habitat of *An. barbirostris *and *An. subpictus *in West Java, whereas in Java hilly areas were preferred by *An. annularis, An. subpictus *and *An. vagus*, as well as possibly *An. indefinitus*, though the latter was not statistically significant. To provide a picture of the findings from this research the topographic/land use relationships for the mosquitoes of West Timor and Java are shown schematically in Figures 2 and 3.

## Discussion

Sub-species or species complexes occur in some anopheline mosquitoes and their vectorial capacity at that level is uncertain. This aspect was beyond the scope of the research reported here, but may be relevant for further research. Although fewer mosquitoes were collected in West Timor than in Java (23% of the total) 81% of these were known to be significant malaria vectors (*An. aconitus, An. barbirostris and An. subpictus*, with the latter very numerous). The abundance and wide occurrence of *An. subpictus *is likely to be a significant contributing factor in West Timor's high incidences of malaria. This is relevant to planning the type of mosquito and thus disease control. Thus if a vector species is concentrated in wetlands in coastal areas, for example *An. subpictus*, there may be the potential to combine methods including predators such as fish with low toxicity larvicides. Where species, such as *An. aconitus*, are widespread such focussed management may not be effective. In that case broad scale larviciding (or adulticiding) may not be cost effective and one option is to encourage the use of protection such as bed nets, screening and wearing long garments. In all situations, providing relevant public information is a cost-effective way to reduce the risk of exposure to disease vector mosquitoes. Such information would be based on knowing the vector habitats and also their diurnal activity patterns, so that humans can avoid contact at particularly risky times.

Also relevant to prioritising control is the result showing several species that are not considered to be very important vectors. These include *An. annularis*, *An. indefinitus *and *An. vagus *(the latter in large numbers in Java) as well as very small numbers of *An. flavirostris*, *An. kochi *and *An. tesselatus*. The results at a species level are consistent with the literature as discussed below.

### Anopheles aconitus

*Anopheles aconitus *had relatively large numbers in hilly-rice field areas in Java and West Timor. Other studies have shown that *An. aconitus *is a malaria vector in rice field areas [8,12,13,19,27-30]. This species is associated with shallow water depths, higher water temperatures, higher acidity and salinity concentrations [20]. *Anopheles aconitus *was confirmed as the main malaria vector in Java and Sumatra [29,31]. It is also a malaria vector in the endemic area of Kokap, Yogyakarta [10,23] and in the Manoreh hills areas [22] and was found also in Bali [30].

### Anopheles annularis

There were relatively few *An. annularis *captured and they were associated with hilly-rice fields, especially in Java. This is similar to other research that found *An. annularis *associated with rice fields, swamps and vegetated pits [28,32]. Noerhadi [33] noted that *An. annularis *was one of the most difficult *Anopheles *species to find. *An. annularis *was also found in Lampung; however it has not been yet confirmed as a malaria vector in the area [32]. Ompusungu *et al *[28] found that *An. annularis *was not a potential malaria vector in Flores, Nusa Tenggara Timor.

### Anopheles barbirostris

*Anopheles barbirostris *was was significant as a coastal specialist in West Timor, whereas it favoured highlands in Java. This is consistent with other research. It has been confirmed as a malaria vector in NTT [34], Sulawesi [35], Bali [30], the Thousand Islands of Jakarta [36], Banten (west Java) and Nias north Sumatra) [37], and Lampung [32]. *An. barbirostris *breeds in swampy rice fields, swamps, lagoons, and fresh water fishponds, neglected fishponds, drainage ditches and rivers [5,28,29,38]. *Anopheles barbirostris *has also been found associated with higher elevations, rice areas, relatively shallow water depths, higher water temperatures, higher acidity and salinity concentrations, and a greater average distance from human habitation [20,39] and the Manoreh hills of Central Java [22].

### Anopheles flavirostris

Only four *An. flavirostris *were captured, and only in the hilly rice fields in Java. It has not been reported as a potential malaria vectors in this area as well as in other areas. For example, it was confirmed as a malaria vector in Sulawesi island in 1949 [38]. In Sikka, Flores, however, the density of this species was too small to transmit malaria [28] and this may well be true in Java, based on this study.

### Anopheles indefinitus

*Anopheles indefinitus *was caught only in hilly-rice field areas in Java. However, it has not been confirmed as a malaria vector in Indonesia. *Anopheles indefinitus *is associated with grassy pools, ponds and ditches [19]. In Sikka, Flores, *An. indefinitus *was found in rice fields and lagoons but in limited numbers only, and in places with low population density [28]. Both the rice field associations and low numbers are consistent with the present research.

### Anopheles kochi

*Anopheles kochi *has mostly been found in Sumatra and Sulawasi [29,37,40] and Halmahera island, North Maluku [41] but it has never been found to be infectious [29]. *Anopheles kochi *is not considered to be a malaria vector in Java.

### Anopheles maculatus

*Anopheles maculatus *was the dominant species in the coastal area of Java in this study. It has a wide distribution in Indonesia including Sumatra, Java, Kalimantan, Bali and Nusa Tenggara Timor islands [29,38,40]. *Anopheles maculatus *is an important species in open hilly areas, in cleared mountain forest areas [19] and in forested hills such as in Central Java [13,22]. Its breeding sites include sunlit puddles in streambeds and along the slow running edges of streams, ditches, rice fields, or close to hilly areas [22,29]. Its presence is also associated with seepage springs, pools and stagnant water in cattle footprints [13].

### Anopheles subpictus

*Anopheles subpictus *was the main *Anopheles *species in coastal areas of West Timor and, in fewer numbers, in hilly-rice fields in Java. 72% of this species was caught in coastal areas. *Anopheles subpictus *has been known to be a malaria vector in Nusa Tenggara Timor Province since 1975 [30,34]. It is widely distributed across Sumatra, Java, Sulawesi, Bali and Nusa Tenggara Timor [10,11,29,40,42,43]. In Sikka-Flores Island (NTT) districts, *Anopheles subpictus *is associated with coastal areas, mainly in mangroves, lagoons, brackish water and river estuaries which have direct sunlight [28,30,42]. Similarly, in the Thousand Island District of Jakarta, *An. subpictus *appeared to be one of the dominant malaria vectors, associated with mangroves and fishponds [36]. Since it is ubiquitous, *An. subpictus *may occur in great density and account for a great amount of malaria [29]. Thus, *An. subpictus *is one of the vectors that needs to be properly controlled to reduce malaria transmission.

### Anopheles sundaicus

Very few *An. sundaicus *were caught, and were entirely in coastal areas, consistent with the findings of others such as Muir [12] who found that on the south coast of Java, artificial fishponds along the edge of relatively calm water were potential *An. sundaicus *sites. *Anopheles sundaicus *is widely distributed in Indonesia's islands of Sumatra, Java, Kalimantan, Bali and Nusa Tenggara Timor, but is not so widely found in Maluku and Papua [8,12,29,31,40,42-44]. It is a significant malaria vector in Indonesia [29,44] and is important in coastal areas [5,8,11,13,19,42,44,45].

### Anopheles tesselatus

There were very few *An. tesselatus *caught in the present study. *Anopheles tesselatus *has been found in Nias island, North Sumatra [37]. It breeds in rice fields, shaded pools, forests, swamps and fresh water fishponds [3,40] and drains with particularly heavy shade [19]. It is not a confirmed malaria vector [41].

### Anopheles vagus

*Anopheles vagus *was one of the most frequently captured mosquitos (37% of the total catch). It was in all topographic settings in West Timor, but it was predominantly a hilly-rice field species in Java. This is consistent with research in Sikka, Flores, in which *An. vagus *was found in almost all types of breeding places: lagoons, rice fields, rivers and stagnant water [28]. *Anopheles vagus *has also been found in the Manoreh hills of Central Java [22]. It occurs widely: in small and open muddy ponds, in animal footprints or in brackish water. Sandosham and Thomas also confirmed that *An. vagus *was endemic everywhere in coastal, hilly and highlands areas [19]. In Java, *An. vagus *was associated with lower elevation fields, deeper and cooler water, and less acidic and saline conditions [20].

Other anophelines that have been recognised to be the main vectors of malaria in Papua such as *Anopheles punctulatus, An. farauti and An. koliensis *[16,17] were not captured in West Timor or Java during this study. This shows that these Australian anophelines may have a distribution restricted to Papua and Maluku islands.

This study has extended knowledge on significant malaria vectors and their distribution in both West Timor and Java. It has shown that the vectors are distributed differently in different areas and topographic settings. *Anopheles annularis *is an important species in both West Timor and Java. *Anopheles maculatus *is a potential malaria vector in highland areas of West Timor but in Java it is a coastal malaria vector. *Anopheles subpictus *was also found in contrasting habitats: in West Timor it was a coastal species whereas in Java it was associated rice field areas. The ubiquitous malaria vector in both areas is *An. vagus *found in all topographical settings in West Timor and in coastal and rice fields areas of Java.

These findings are important for malaria management program to help design malaria vector control programs relevant to local characteristics including the vectors and their activity patterns.

## Conclusion

The results from this study show that each *Anopheles *species occupied different topographical settings. That there were four highly significant vectors of malaria with different distributions by location and topographic type is information that can be used to inform and prioritize mosquito and malaria management. Thus, in West Timor, *An. subpictus *is probably the most important species found only (in this research) in the coastal areas with some *An. barbirostris. *In Java, *An. aconitus *is important and ubiquitous, though more frequent in hilly areas, as is *An. subpictus *(though with fewer numbers); in highland areas, *An. barbirostris *is the main problem species.

This information is potentially useful in the mosquito control aspect of malaria management focussing attention on the habitats that harbour the more serious or more numerous vectors of assemblages that together may lead to high incidences of malaria. As discussed above, the management combinations may be planned for a variety of situations: for localized habitats direct intervention by larviciding or creating predator reservoirs may be accost effective option, For widespread species then protection (e.g., bed nets, screening) is likely to be cost-effective,. The role of public information is also important so that people can plan activities to minimize risk of contact with disease vector mosquitoes. This needs information not only on the vector habitats but also on diurnal mosquito activity patterns, which is the subject of another paper.

## Competing interests

The authors declare that they have no competing interests.

## Authors' contributions

ED carried out all the field work and most of the data analysis. CW provided entomological and editorial advice. PD participated in the design and implementation of the research and provided editorial assistance. NS participated in the design and implementation of the research and provided editorial assistance. MD participated in the design and implementation of the research and provided advice on analysis.

All authors have read and approved the final manuscript.
